# Correlation Between Facial Asymmetry and Maxillary Sinus Size or Volume Using Dental Cone-Beam Computed Tomography Images

**DOI:** 10.7759/cureus.80118

**Published:** 2025-03-05

**Authors:** Mayuko Takeda, Keiko Kujirai, Masahiro Takahashi, Reina Hatanaka, So Koizumi, Yu Hikita, Tetsutaro Yamaguchi

**Affiliations:** 1 Department of Orthodontics, School of Dentistry, Kanagawa Dental University, Yokosuka, JPN

**Keywords:** cone-beam computed tomography, facial asymmetry, facial growth, maxillary sinus, skeletal morphology

## Abstract

Objectives

This study investigated correlations between facial asymmetry and left-right differences in maxillary sinus size and volume, as well as their relationships with skeletal morphology.

Methods

The participants were 154 Japanese adults (56 men, 98 women) aged 18 to 38 years (mean age, 24.1 ± 5.3 years). Cone-beam computed tomography (CBCT) images were analyzed using InVivo™ 6 software (Anatomage, Inc., San Jose, CA, USA). Facial asymmetry was assessed at six landmarks: orbitale, condylion, and gonion (all bilateral); anterior nasal spine; deepest point in bony concavity (B point); and menton (at midline). Maxillary sinus height, width, length, and volume were measured. Participants were classified into skeletal classes (I, II, III) and vertical growth patterns (horizontal, average, vertical). Spearman’s rank correlation coefficient was used to assess the relationships of these six facial asymmetry landmarks with differences in maxillary sinus linear measurements and volume.

Results

The facial asymmetry index was positively correlated with maxillary sinus length in the condylion region and height in the gonion region. As left-sided facial deviation increased, the right maxillary sinus became larger; conversely, as right-sided facial deviation increased, the left maxillary sinus became larger. Thus, the maxillary sinus on the nondeviated side tends to be larger. Vertical facial growth patterns showed greater maxillary sinus height and volume in the vertical growth group than in the average growth group.

Conclusions

Asymmetry in specific facial regions may be associated with region-specific variations in maxillary sinus morphology, and vertical skeletal patterns may influence maxillary sinus development. These findings offer insights into the relationship between facial asymmetry and maxillary sinus structure.

## Introduction

The maxillary sinus, located in the center of the maxilla, is the largest of the paranasal sinuses. It has a pyramidal shape, with the zygomatic process at its apex. The upper wall forms the floor of the orbit, while the inner wall is part of the lateral wall of the nasal cavity. The alveolar process and hard palate form the floor, and the posterior wall is adjacent to the pterygopalatine and infratemporal fossae [1]. Maxillary sinus growth occurs in the central part of the face and plays an important role in the development of facial contours and dentition [2]. The maxillary sinus begins to develop around the third month of life and continues to expand outward and downward until adulthood [3]. As it grows, the floor of the sinus extends into the posterior alveolar process, forming an alveolar recess, and the apices of the teeth protrude into the sinus [4]. Because of these characteristics, the maxillary sinus can serve as an anatomical limiting factor for tooth movement during orthodontic treatment; tooth movement through the sinus may result in excessive tooth tipping, root resorption, or sinus perforation [4].

In orthodontic treatment for patients with jaw deformities, surgical orthodontic intervention combined with orthognathic surgery is used to improve facial appearance and correct occlusal abnormalities [5]. The Le Fort I osteotomy, a common technique in orthognathic surgery, involves cutting along the maxillary sinus; thus, it is important to have a detailed understanding of maxillary sinus morphology. Previous studies have identified changes, such as a reduction in total sinus volume and an increase in the thickness of the lower maxillary sinus wall with age after adulthood, although the timing of these changes is unclear [6]. Additionally, orthodontic anchor screws (mini screws), frequently inserted between the second premolar and first molar in the maxillary alveolar region for anchorage, carry a risk of accidental perforation of the maxillary sinus at the insertion site [7]. Therefore, adequate knowledge of the maxillary sinus's morphology and anatomy is essential for orthodontic treatment planning and avoiding complications. For anchor screw placement, understanding its position and structure ensures safe and successful fixation. In surgical orthodontics, detailed knowledge minimizes intraoperative and postoperative risks.

Thus far, multiple researchers have examined the relationships of maxillary sinus morphology, such as height, width, length, and area, with malocclusion classification and maxillofacial skeletal morphology using orthopantomography and lateral cephalometric radiographs [8-10]. However, because the maxillary sinus is a three-dimensional (3D) structure, conventional two-dimensional (2D) X-ray measurements can lead to errors [9]. The borders of the maxillary sinus often overlap with deep regions of the nasomaxillary complex in these images, hindering accurate measurements [9]. Additionally, the width and diameter of the maxillary sinus cannot be accurately assessed with 2D images [9]. Therefore, the use of 2D images has important limitations when attempting to obtain accurate measurements of the maxillary sinus [11].

Cone-beam computed tomography (CBCT) is a technique for maxillofacial imaging, first reported in the literature by Mozzo et al. [12] and Oz et al. [13]. Because of the limitations of 2D imaging, studies in recent decades have utilized CBCT to investigate the relationship between maxillary sinus morphology and skeletal malocclusion. Okşayan et al. [14] classified vertical maxillofacial skeletal morphology into three groups, high angle, low angle, and normal angle, based on the angle between the sella (S)-nasion (N) plane and the gonion (Go)-gnathion line; they subsequently evaluated the relationship between maxillary sinus dimensions (height, width, and length) and volume. Similarly, Atul Kumar et al. [11] categorized craniofacial patterns into three groups, horizontal, average, and vertical, based on the Frankfort mandibular plane angle (FMA) formed by the Frankfort horizontal plane and the mandibular plane. They examined the relationships of skeletal base height, maxillary sinus width and length, and alveolar bone height with maxillary sinus width, depth, height, and volume.

Several studies have explored the relationships of anteroposterior and vertical maxillofacial skeletal morphology with 3D maxillary sinus morphology. Shrestha et al. [15] found that individuals with skeletal class II had a significantly larger maxillary sinus volume relative to those with skeletal class III. Among the vertical skeletal morphology groups, no significant differences were observed; however, the high-angle group tended to have a larger maxillary sinus volume. Abate et al. [16] also investigated maxillary sinus morphology using CBCT images. They found no correlation between the anteroposterior skeletal relationship, classified by ANB angle (i.e., the angle formed by point A, N, and point B), and maxillary sinus volume across the three groups. However, maxillary sinus length and volume were significantly greater among individuals with a protruding or receding maxilla, classified by the SNA angle (i.e., the angle formed by S, N, and point A), relative to the normal group. Okşayan et al. [14] evaluated maxillary sinus volume and dimensions across different vertical facial growth patterns. Although some controversy remains, these studies suggest that a correlation exists between skeletal morphology and maxillary sinus characteristics. In addition to vertical and anteroposterior craniofacial morphology issues, facial asymmetry and jaw deviation can cause both functional and aesthetic problems, which may have negative impacts on psychological and social development [17]. Facial asymmetry can be categorized into three main types: congenital, which occurs before birth; developmental, which arises during growth without an obvious etiology; and acquired, which results from injury or disease. In many cases, the cause remains unknown. Regarding the relationship between jaw deviation and the maxillary sinuses, Basdra et al. [2] reported a case in which bone deposition of unknown origin in the right maxillary sinus led to compensatory enlargement of the left maxillary sinus. This enlargement caused expansion of the left dental arch, resulting in asymmetrical maxillary dental arches and a left crossbite, which was thought to contribute to the asymmetry. Moreover, Ahn et al. [18] investigated the relationship between asymmetry patterns and nasomaxillary complex morphometrics in patients with skeletal class III facial asymmetry; they found no difference in maxillary sinus volume between the right and left sides, regardless of facial asymmetry. Teodorescu et al. [19] studied the morphological features of the upper airway cavities in 41 patients aged seven to 37 years with facial asymmetry, assessed by posteroanterior cephalography. Most of the patients had narrow airways on the underdeveloped side of the face, as well as large sinuses. Teodorescu et al. [19] noted that it remains unclear whether the changes in airway and sinus morphology were caused by facial asymmetry or if they were compensatory. Although some studies have explored the relationship between facial asymmetry and the maxillary sinus, the nature of any correlation remains uncertain.

To better understand the relationship between jaw deviation and maxillary sinus morphology, this study explored links between the degree of jaw deviation (facial asymmetry) and the size and volume of the maxillary sinus, measured in three dimensions using CBCT. Our null hypothesis was that facial asymmetry is not correlated with maxillary sinus size or volume.

## Materials and methods

This retrospective study protocol was approved by the Ethics Committee of Kanagawa Dental University (Approval Number: 641, Approval Date: February 26, 2020; Approval Number: 911, Approval Date: March 13, 2023; Approval Number: 923, Approval Date: April 17, 2023; Approval Number: 1047, Approval Date: November 20, 2024).

Participants

Because this was an exploratory study and no similar studies have been conducted in the past, no a priori sample size calculations were performed. This study included 154 Japanese patients (56 men and 98 women; mean age, 24.1 years) between the ages of 18 and 38 years who visited the orthodontic clinic of Kanagawa Dental University Hospital, Yokosuka, Japan, from February 2020 to October 2023. The inclusion criterion was an age of 18 years or older. Exclusion criteria were congenital diseases, a history of orthodontic treatment or orthognathic surgery, and CBCT images of poor quality. The CBCT data used in this study were not specifically acquired for this research. The study adhered to the Declaration of Helsinki and followed current standards for reporting observational studies in epidemiology (i.e., the STROBE checklist). Written informed consent was obtained from all patients prior to treatment.

Data acquisition and 3D landmarks

CBCT data were obtained using a cone-beam X-ray system (KaVo 3DeXam, KaVo, Biberach, Germany). All data were stored in digital imaging and communications in medicine (DICOM) format and reconstructed as 3D images using the imaging software InVivo™ 6 (Anatomage, Inc., San Jose, CA, USA) for further processing and analysis. All measurements were assessed using InVivo™ 6. Eleven anatomical 3D landmarks were selected for analysis, following the procedure outlined in a previous study [20].

Landmarks and reference angles for analyzing anteroposterior and vertical maxillofacial skeletal morphology were selected based on procedures followed in previous studies [11,21].

Maxillary sinus measurements

**Figure 1:**
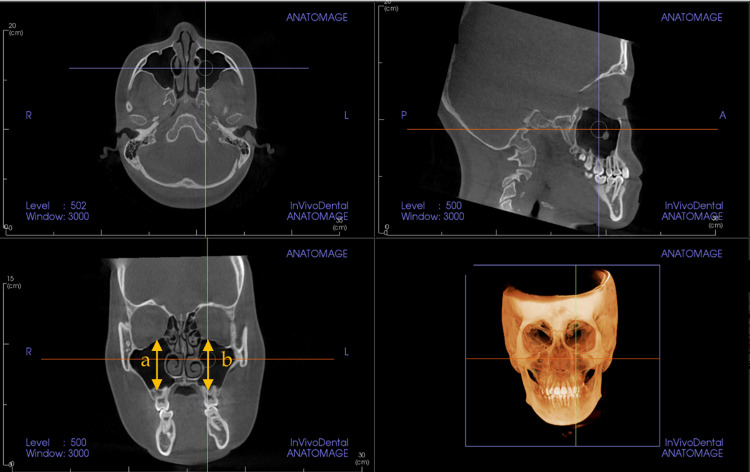
Cone-beam computed tomography (CBCT) images used to measure maxillary sinus height. The longest distance from the lowest point of the sinus floor to the highest point of the sinus roof was assessed using a coronal CBCT image. a: right maxillary sinus height; b: left maxillary sinus height

**Figure 2:**
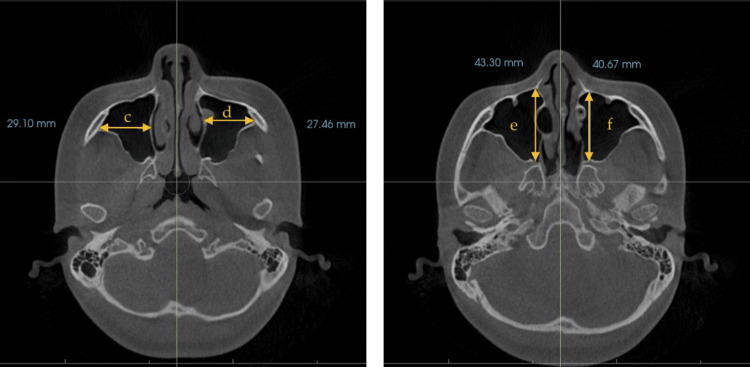
Measurement of maxillary sinus length and width using an axial cone-beam computed tomography (CBCT) image. The figure on the left is A, and the figure on the right is B. A: measurement of maxillary sinus width using an axial CBCT image. This parameter comprised the longest distance from the most medial wall of the sinus to the most lateral wall. c: right maxillary sinus width; d: left maxillary sinus width B: measurement of maxillary sinus length using an axial CBCT image. This parameter comprised the longest distance from the most anterior point to the most posterior point of the medial wall. e: right maxillary sinus length; f: left maxillary sinus length

**Figure 3:**
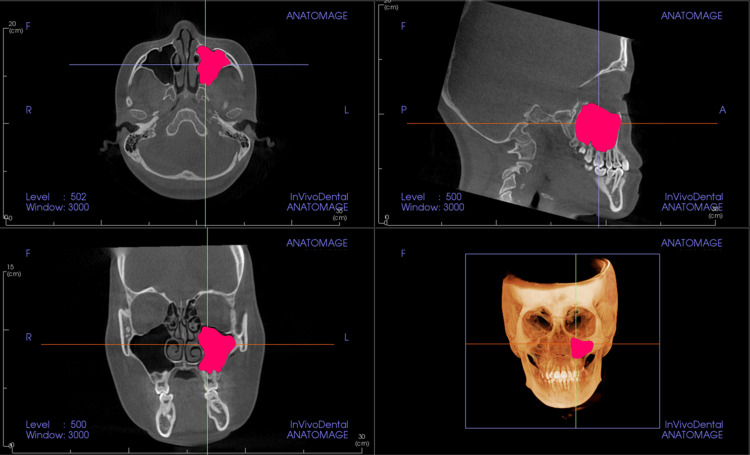
3D volume reconstruction of the maxillary sinus using cone-beam computed tomography images. The pink area represents the left maxillary sinus. Maxillary sinus volume measurements were taken for both the left and right sides.

**Figure 4:**
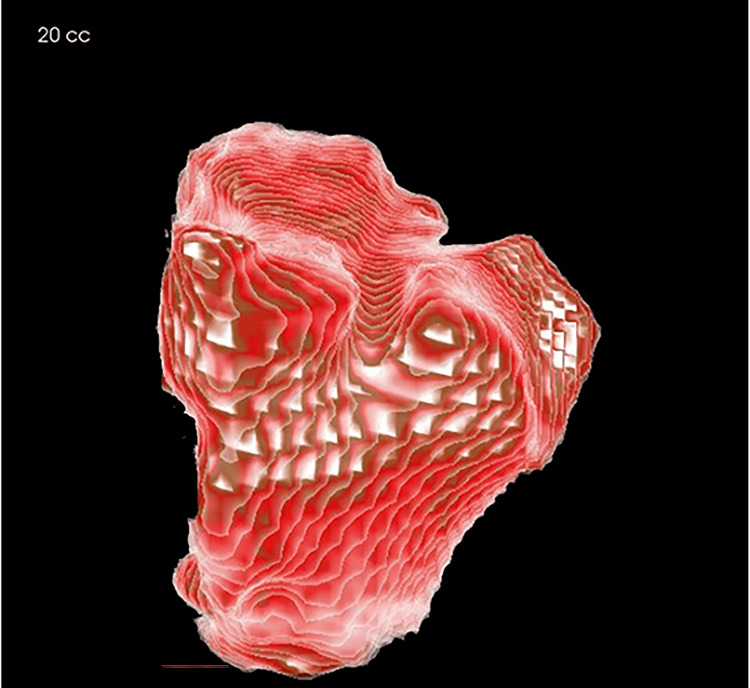
3D volumetric measurement of the maxillary sinus. This figure shows the left maxillary sinus volume measurement.

The size of the maxillary sinus was measured at its maximum height on both sides (Figure 1). The maximum width was measured from the inner wall to the outer wall on a coronal image (Figure 2A), and the maximum anteroposterior diameter was measured from the anterior to the posterior wall of the maxillary sinus on a coronal image (Figure 2B). Additionally, the volume of the maxillary sinus was calculated (Figure 3). Figure 4 shows the left maxillary sinus extracted for volume measurement. In the InVivo™ 6 software, linear measurements were recorded in millimeters, and volumes were measured in cubic millimeters (mm³). All measurements were performed by a single researcher (M.T.) using CBCT images. To assess measurement error, 20 CBCT images were randomly selected, and measurements were repeated at two-week intervals under the same conditions using Dahlberg’s formula [22].

Analysis of facial asymmetry

Facial asymmetry was assessed using the facial asymmetry index and the facial asymmetry index on midsagittal landmarks, as proposed by Cao et al. [20]. The facial asymmetry index evaluates the asymmetry of the orbitale (Or), gonion (Go), and condylion (Co) points on both sides of the face, whereas the midsagittal index assesses asymmetry at the anterior nasal spine (ANS), menton (Me), and the deepest point in the bony concavity (B point) along the facial midline. Asymmetry was evaluated at a total of six points: Or, Go, Co, ANS, Me, and B point.

Facial asymmetry index

**Figure 5:**
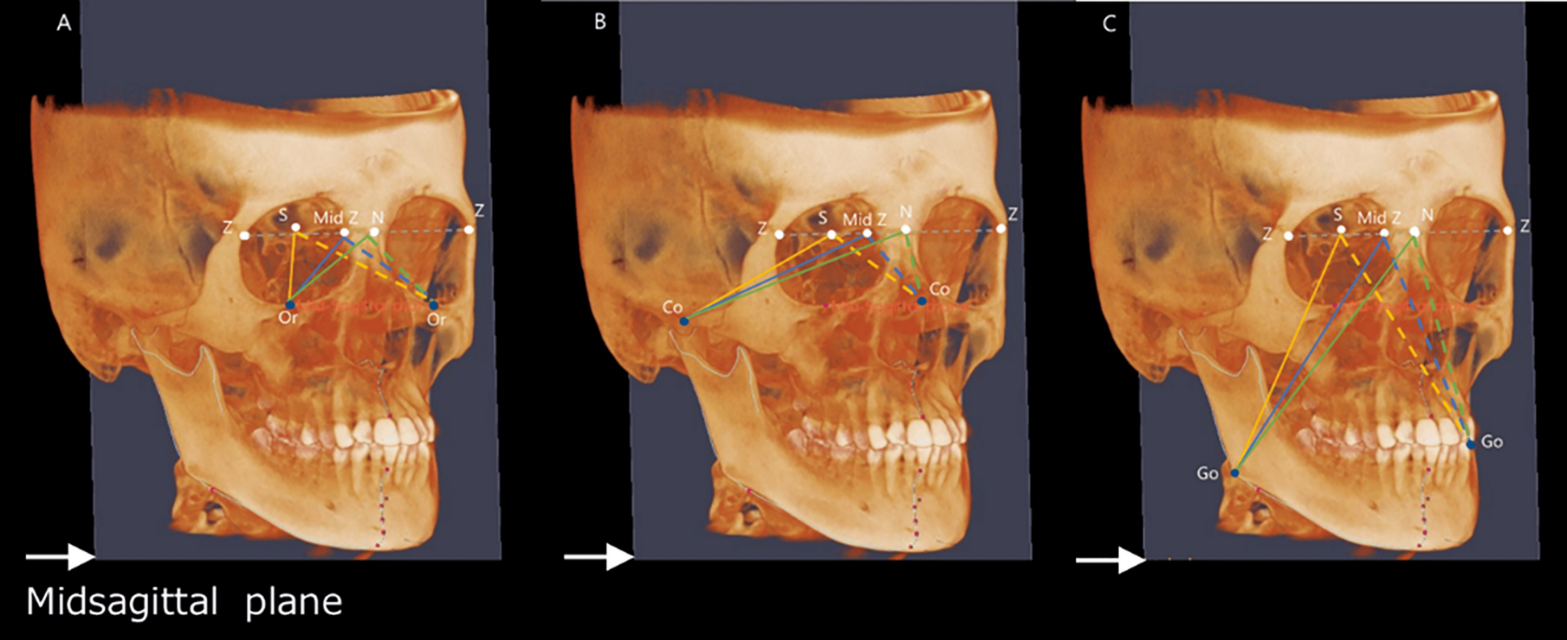
Evaluation of each facial asymmetry index using cone-beam computed tomography images. A: distance measurement from each reference point to Or; B: distance measurement from each reference point to Co; C: distance measurement from each reference point to Go. Right side: solid line; left side: dotted line; S (sella): center of the pituitary fossa; N (nasion): nasofrontal suture at midline; Mid Z (midpoint of Z): midpoint of the line between both Z points; Z point: the most inferior point on the zygomaticomaxillary suture; Or (orbitale): the lowest points of the orbital rim; Co (condylion): the most superior point of the condyles; Go (gonion): the most inferior and posterior points at the angles of the mandible

**Figure 6:**
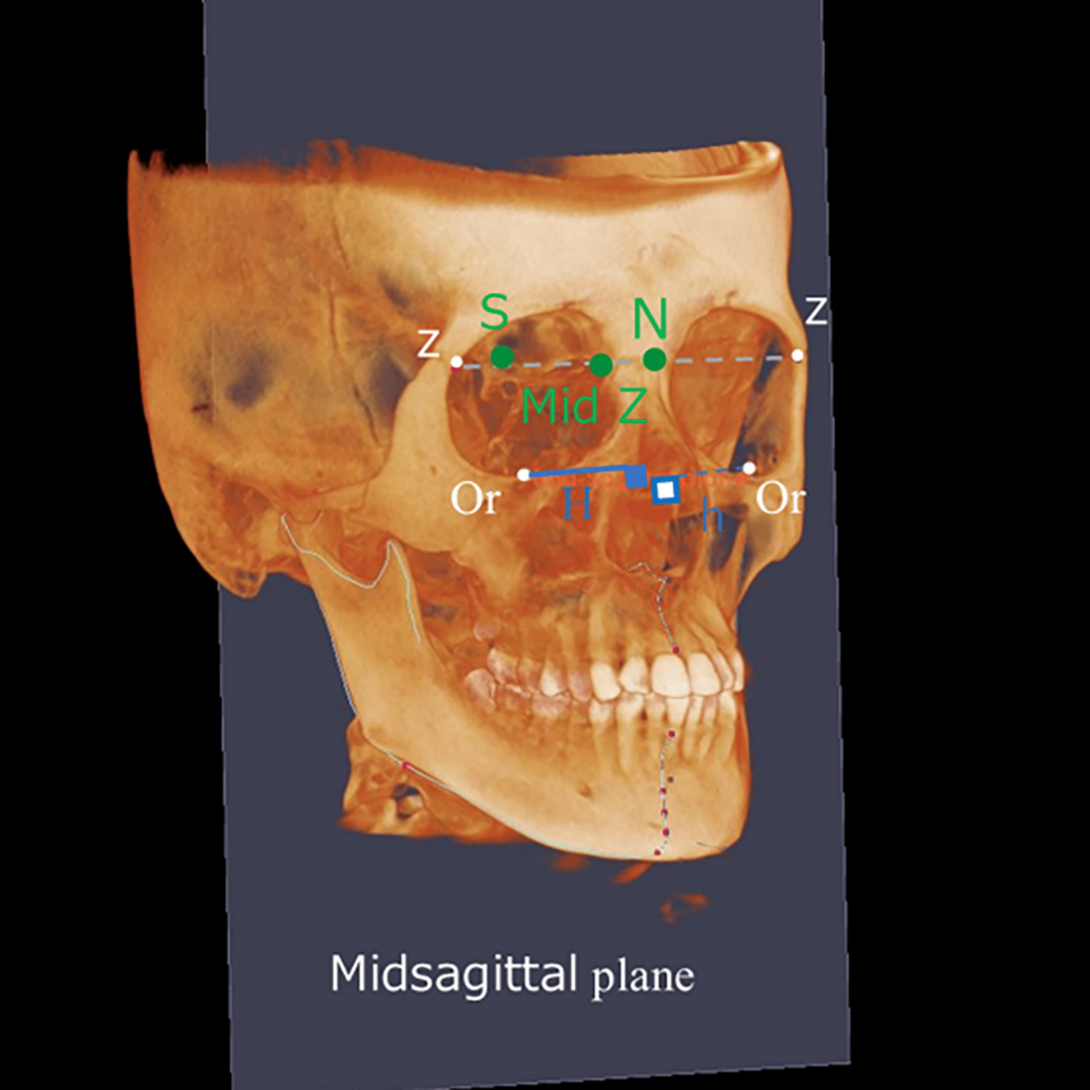
The midsagittal plane composed of N, S, and Mid Z, along with the H-line using cone-beam computed tomography images. H: perpendicular line from the right Or to the midsagittal plane; h: perpendicular line from the left Or to the midsagittal plane

To calculate the asymmetry index for the paired landmarks Or, Co, and Go, the distances from N, S, and the midpoint of the line between the most inferior points on the zygomaticomaxillary suture (Mid Z) to the left and right Or, Co, and Go points were measured (Figure 5). The facial asymmetry index is determined by calculating the sum of the squared differences in these distances between the left and right sides (right-side value minus left-side value) for each point (Or, Co, Go). The square root of this sum constitutes the facial asymmetry index. The k value, used to identify the side of deviation, is calculated by comparing the lengths of the perpendicular lines (H: right side, h: left side) from each point to the midsagittal plane, which serves as the reference (Figure 6). The side with the longer perpendicular line indicates the direction of deviation. If the right-side H (i.e., the perpendicular line from the right Or to the midsagittal plane) is larger than the left-side h (the perpendicular line from the left Or to the midsagittal plane), the k value is −1. If the right-side H is smaller than the left-side h, the k value is +1. This value is then multiplied by the previously calculated facial asymmetry index. The index will show negative values for right-sided deviation and positive values for left-sided deviation. Figure 6 illustrates the perpendicular lines H and h from Or to the midsagittal plane. If the left and right points (Or, Co, Go) are 3D symmetrical, the facial asymmetry index will be 0. An increase in this value indicates greater asymmetry.

Using the measurement point Or as an example, the formula is as follows (1): $\text{Facial asymmetry index} = k \sqrt{(A - a)^2 + (B - b)^2 + (C - c)^2}$ (1)

In Equation (1), right-side deviation if H > h, k = −1 and left-side deviation if H < h, k = +1. A: nasion to orbitale (right side); a: nasion to orbitale (left side); B: sella to orbitale (right side); b: sella to orbitale (left side); C: mid Z to orbitale (right side); and c: mid Z to orbitale (left side).

**Figure 7:**
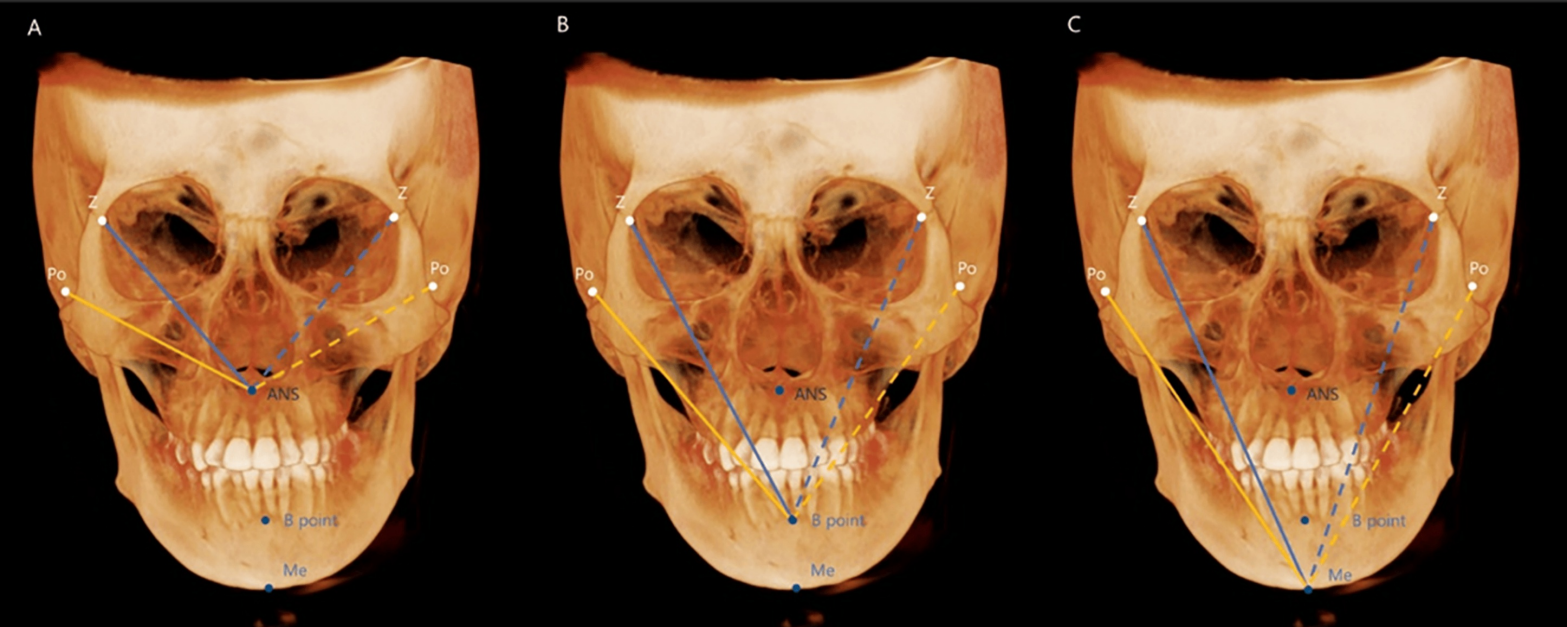
Evaluation of the facial asymmetry index for midsagittal landmarks using cone-beam computed tomography images. A: distance measurement from each reference point to ANS; B: distance measurement from each reference point to B point; C: distance measurement from each reference point to Me. Right side: solid line; left side: dotted line; ANS: anterior nasal spine; B point: the deepest point in the bony concavity in the mandibular midline; Me (menton): the lowest border of the mandible; Z point: the most inferior point on the zygomaticomaxillary suture; Po (porion): the most inferior point of the external auditory meatus

This formula was used to calculate the Or, Go, and Co facial asymmetry indices. To calculate the facial asymmetry index for the midsagittal landmarks (ANS, B point, and Me), the left and right distances from the Z points and porion (Po) to the ANS, B point, and Me were measured (Figure 7). The sum of squared differences between the left and right distances (right-side value minus left-side value) from the Z point and Po to each point (ANS, B point, Me) is calculated; the square root of this sum constitutes the facial asymmetry index for midsagittal landmarks. The k value determines the side of deviation: if the sum of the differences is less than 0, the k value is −1, indicating right-sided deviation; if it is greater than 0, the k value is +1, indicating left-sided deviation. Using the ANS as an example, the formula is as follows:

Equation (2), (3), and (4):

$\text{Facial asymmetry index on midsagittal landmarks} = k \sqrt{(A - a)^2 + (B - b)^2}$ (2)

$\text{If } (A - a) + (B - b) &lt; 0, \quad k = -1 \; (\text{right-sided deviation})$ (3) 

$\text{If } (A - a) + (B - b) > 0, \quad k = +1 \; (\text{left-sided deviation})$ (4)

Where A: Z point to ANS (right side); a: Z point to ANS (left side); B: porion to ANS (right side); and b: porion to ANS (left side). Based on this formula, the facial asymmetry indices for midsagittal landmarks were calculated for the ANS, B point, and Me.

Analysis of side faces

Using cephalograms constructed from CBCT data, the skeletal anteroposterior relationship was classified into three skeletal classes based on the ANB angle, in accordance with the method of Deguchi et al. [21]: skeletal class I, 0 ≦ ANB < 6°; skeletal class II, ANB ≧6°; and skeletal class III, 0 > ANB. The vertical and anteroposterior maxillofacial skeletal morphology of all participants, including both the jaw deviation and nondeviated groups, were classified into three categories. Vertical maxillofacial skeletal morphology was classified according to craniofacial pattern using the method of Atul Kumar et al. [11], defined by the FMA as follows: FMA < 22°, horizontal group; 22° ≦ FMA < 28°, average group; and 28° ≦ FMA, vertical group.

Statistical analysis

Statistical analysis was performed using IBM SPSS Statistics for Windows, Version 25 (Released 2017; IBM Corp., Armonk, New York, United States). Normality and homogeneity of variance were assessed using the Shapiro-Wilk and Levene tests. Spearman’s rank correlation coefficient was calculated to examine the relationship between maxillary sinus size and the asymmetry index. For multiple comparisons, Bonferroni correction was applied, with the significance threshold set at p < 0.002. The Kruskal-Wallis test was used to compare maxillary sinus linear measurements and volumes among the three groups based on classifications of anteroposterior skeletal relationships and vertical maxillofacial skeletal morphology. Additionally, the Mann-Whitney U test was performed to compare differences between each pair of groups. The significance threshold was set at p < 0.05, indicating 5% probability. Spearman’s rank correlation coefficient was used to examine the relationship between maxillary sinus size and the asymmetry index; statistical significance was determined via division of 5% probability by the total number of tests (24). This resulted in a Bonferroni-corrected significance threshold of p < 0.002, adjusting for multiple comparisons. Before analyzing the correlations of maxillary sinus size with the asymmetry index and asymmetry index for midsagittal landmarks, patients within ±1 standard deviation (SD) of the mean for each asymmetry index were classified into a subgroup without facial asymmetry. The Mann-Whitney U test was then performed to confirm that there were no significant differences in maxillary sinus size (height, width, length, and volume) between the left and right sides in the subgroup. The significance threshold was set at p < 0.05, indicating 5% probability. Intraclass correlation coefficients (ICCs) were calculated to ensure the reliability of each measurement, and the Dahlberg formula was applied to verify the validity of the measurement error range.

## Results

Measurement errors ranged from 0.16 to 0.22 mm for linear measurements and 0.11 to 0.20 mm³ for volume measurements using CBCT. The facial asymmetry index ranged from 0.26 to 0.66, whereas the facial asymmetry index for midsagittal landmarks ranged from 0.18 to 0.52. Additionally, ICC values for interobserver and intraobserver reliability were greater than 0.85, indicating acceptable reliability for all measurements (Table 1).

**Table 1:** Assessments of intraobserver reproducibility. L: left side; R: right side; Or: the lowest points of the orbital rim; Co: the most superior point of the condyles; Go: the most inferior and posterior points at the angle of the mandible; ANS: anterior nasal spine; B point: the deepest point in the bony concavity in the mandibular midline; Me: the lowest border of the mandible; MSH: maxillary sinus height; MSL: maxillary sinus length; MSW: maxillary sinus width; MSV: maxillary sinus volume; ICC: intraclass correlation coefficient

Variable	Dahlberg	ICC
Facial asymmetry index		
Or	0.66	0.89
Co	0.35	0.96
Go	0.51	0.99
Facial asymmetry index on midsagittal landmarks		
ANS	0.37	0.96
B point	0.53	0.96
Me	0.52	0.90
Maxillary sinus		
MSH (R)	0.33	0.99
MSH (L)	0.18	0.99
MSW (R)	0.21	0.99
MSW (L)	0.16	0.99
MSL (R)	0.22	0.99
MSL (L)	0.22	0.99
MSV (R)	0.11	0.99
MSV (L)	0.20	0.99

The ICC values for cephalometric measurements were also > 0.88, confirming high reliability of the measured values (Table 2). Furthermore, the measurement error calculated using Dahlberg’s equation ranged from 0.34° to 0.51°, which is considered acceptable.

**Table 2:** Assessments of intraobserver reproducibility in cephalometric analysis. SNA: angle formed by S, N, and point A; SNB: angle formed by S, N, and point B; ANB: angle formed by point A, N, and point B; FMA: Frankfort mandibular plane angle; ICC: intraclass correlation coefficient

	Dahlberg	ICC
SNA	0.51	0.88
SNB	0.34	0.96
ANB	0.36	0.95
FMA	0.34	0.98

The medians and interquartile ranges of maxillary sinus dimensions (height, width, length, and volume) for all 154 participants are presented in Table3.

**Table 3:** Descriptive statistics for maxillary sinus measurements. L: left side; R: right side; MSH: maxillary sinus height; MSW: maxillary sinus width; MSL: maxillary sinus length; MSV: maxillary sinus volume

Variables	Right side			Left side			Difference (R–L)		
N = 154	Q1(25%Quartiles)	Q2(Median)	Q3(75%Quartiles)	Q1(25%Quartiles)	Q2(Median)	Q3(75%Quartiles)	Q1(25%Quartiles)	Q2(Median)	Q3(75%Quartiles)
MSH (mm)	36.6	40.3	43.3	36.4	40.0	43.1	-1.6	0.2	2.8
MSW (mm)	27.5	29.5	31.5	26.7	28.7	31.1	-0.5	0.8	2.4
MSL (mm)	36.8	38.9	41.1	36.5	38.7	40.7	-0.8	0.3	1.7
MSV (mm3)	14.2	17.7	21.2	14.0	17.3	19.6	-0.9	0.4	2.4

The results of statistical analyses regarding the correlations of left-right differences in maxillary sinus size with the facial asymmetry index and facial asymmetry index on midsagittal landmarks are shown in Table 4. Statistically significant positive correlations were observed between the facial asymmetry index (Go) and maxillary sinus height in the Go area, as well as between the facial asymmetry index (Co) and maxillary sinus length in the Co area.

**Table 4:** Results of the Spearman rank correlation analysis between facial asymmetry index and maxillary sinus size. Or: orbitale; Go: gonion; Co: condylion; ANS: anterior nasal spine; B point: the deepest point in the bony concavity in the mandibular midline; Me (menton): the lowest border of the mandible. MSHD: maxillary sinus height difference; MSLD: maxillary sinus length difference; MSVD: maxillary sinus volume difference; MSWD: maxillary sinus width difference. *Indicates a significant correlation, a < 0.002 (Bonferroni-corrected)

ALL N=154		Asymmetry Index (Or)	Asymmetry Index (Go)	Asymmetry Index (Co)	Asymmetry Index on Midsagittal Plane (ANS)	Asymmetry Index on Midsagittal Plane (B point)	Asymmetry Index on Midsaggital Plane (Me)
MSHD (R-L)	Correlation Coefficient	0.13	0.26	0.18	-0.18	0.07	0.13
	P-value	0.12	< 0.01*	0.03	0.03	0.41	0.10
MSWD (R-L)	Correlation Coefficient	-0.06	0.12	-0.01	< 0.01	0.03	0.05
	P-value	0.43	0.14	0.89	0.99	0.69	0.52
MSLD (R-L)	Correlation Coefficient	0.15	0.16	0.28	-0.16	-0.13	-0.11
	P-value	0.07	0.04	< 0.01*	0.04	0.11	0.18
MSVD (R-L)	Correlation Coefficient	-0.02	0.02	-0.09	0.17	0.09	0.12
	P-value	0.82	0.77	0.28	0.04	0.28	0.14

The results of the comparisons between groups based on anteroposterior skeletal relationships and vertical maxillofacial skeletal morphology are presented in Tables 5, 6.

**Table 5:** Results of statistical analyses concerning right and left sinus variables based on Kruskal-Wallis and Mann-Whitney U tests between groups based on anteroposterior skeletal relationships. Threshold for statiscal significance is P<0.05

		Skeletal class I			Skeletal class II			Skeletal class III		Skeletal class I vs. II	Skeletal class II vs. III	Skeletal class III vs. I
		n = 78			n=38			n = 38				
Variables	25%	Median	75%	25%	Median	75%	25%	Median	75%	P value	P value	P value
Right maxillary sinus height	36.6	40.2	43.0	36.5	40.2	42.5	37.4	41.8	44.6	0.67	0.29	0.53
Right maxillary sinus width	27.4	29.5	31.5	27.3	29.8	31.3	27.5	29.3	33.0	1.00	0.80	0.75
Right maxillary sinus length	36.8	39.1	41.2	37.0	39.1	42.0	36.5	38.6	40.2	0.98	0.41	0.33
Right maxillary sinus volume	14.5	17.6	21.9	14.4	17.9	21.3	13.1	16.3	20.5	0.87	0.87	0.29
Left maxillary sinus height	36.5	40.0	43.0	36.6	39.3	42.2	36.0	40.4	44.5	0.51	0.46	0.91
Left maxillary sinus width	26.6	28.9	30.9	25.8	28.7	31.3	27.1	29.0	32.0	0.86	0.45	0.46
Left maxillary sinus length	37.1	39.0	40.1	36.4	38.5	40.7	36.0	38.0	41.0	0.42	0.68	0.16
Left maxillary sinus volume	14.6	17.6	19.9	13.1	19.4	17.3	12.8	17.2	21.0	0.27	0.93	0.41

**Table 6:** Result of statistical analyses concerning right and left sinus variables based on Kruskal-Wallis and Mann-Whitney U tests between groups based on vertical maxillofacial skeletal morphology. **p<0.01, ***p<0.001

	Vertical Group (n=93)			Average Group (n=47)			Horizontal Group (n=14)			Vertical vs. Average	Average vs. Horizontal	Horizontal vs. Vertical
Variables	25%	Median	75%	25%	Median	75%	25%	Median	75%	P-value	P-value	P-value
Right maxillary sinus height	38.9	40.9	44.3	34.1	37.8	42.4	38.7	40.6	42.6	< 0.01***	0.12	0.60
Right maxillary sinus width	27.5	29.7	32.2	27.1	28.5	30.8	27.9	30.5	34.3	0.27	0.14	0.39
Right maxillary sinus length	37.4	39.1	41.2	36.3	38.1	40.1	36.3	40.2	43.4	0.06	0.19	0.60
Right maxillary sinus volume	14.7	19.0	21.8	13.1	15.6	18.9	16.1	19.9	22.9	< 0.01***	0.02	0.37
Left maxillary sinus height	37.1	40.7	44.1	35.1	38.4	41.3	39.3	40.0	43.8	< 0.01***	0.02	0.65
Left maxillary sinus width	26.0	28.7	31.2	27.1	28.5	30.8	28.0	29.7	33.5	0.71	0.10	0.13
Left maxillary sinus length	36.8	38.8	40.8	36.1	38.2	40.3	42.8	37.8	39.9	0.15	0.06	0.88
Left maxillary sinus volume	14.6	17.6	19.8	12.5	15.9	18.6	14.6	17.8	21.1	< 0.01***	0.05	0.88

Concerning anteroposterior skeletal relationships, the Kruskal-Wallis test showed no statistically significant differences in the height, width, length, or volume of the right or left maxillary sinus among the three groups: skeletal class I, skeletal class II, and skeletal class III. Additionally, comparisons between groups using the Mann-Whitney U test did not reveal any statistically significant differences across all measures.

The Kruskal-Wallis test showed statistically significant differences in the height and volume of the right and left maxillary sinuses among the three groups: horizontal, average, and vertical. Furthermore, the Mann-Whitney U test revealed that the vertical group had significantly greater maxillary sinus height and volume compared with the average group on both the right and left sides.

## Discussion

The lateral relationships of skeletal anteroposterior morphology, vertical maxillofacial skeletal morphology, and maxillary sinus morphology have been examined [8,11,14,18,23-25]. However, research on the relationship between frontal facial asymmetry and maxillary sinuses is limited. Basdra et al. [2] provided a case report, while Ahn et al. [18] investigated correlations between asymmetry patterns and morphometric measurements of the maxillary sinus, nasal cavity, and nasomaxillary complex in skeletal class III individuals using CBCT images. In the present study, we explored the relationships between facial asymmetry and the internal structures of the nasomaxillary complex, specifically, the height, width, length, and volume of the maxillary sinus, using 3D linear measurements. To our knowledge, this is the first study to use CBCT images to measure facial asymmetry, maxillary sinus size, and maxillary sinus volume, and to investigate correlations between the degree of asymmetry at specific facial locations and left-right differences in maxillary sinus linear measurements. Significant positive correlations were observed between the asymmetry index and maxillary sinus depth at Co, as well as between the asymmetry index and maxillary sinus height at Go. This indicates that the nondeviated side tends to have a larger maxillary sinus than the deviated side; conversely, the deviated side tends to have a smaller sinus than the nondeviated side. The null hypothesis, which stated that facial asymmetry is not correlated with maxillary sinus size or volume, was rejected. The mandibular condyle head, located in the mandibular fossa, is anatomically closely connected to the cranial base. Based on this anatomical relationship, it has been suggested that asymmetry in the mandibular condyle head contributes to distortion of the maxillary body. Chen et al. [26] reported that the length of the skull base was greater on the nondisplaced side than on the displaced side among individuals with facial asymmetry. They found that in asymmetric individuals with chin deviation, relative overgrowth of the mandibular condyle head on the unaffected side causes mandibular structures to grow anteriorly, inferiorly, and contralaterally. This growth leads to adaptive changes in the maxilla, dentition, and alveolar bone, which follow the asymmetric growth of the mandible. There are various causes of maxillofacial asymmetry; however, asymmetric growth of the mandibular condyle head has been shown to induce asymmetry in mandibular development, which subsequently leads to asymmetric maxillary growth. The significant positive correlation between the asymmetry index and maxillary sinus depth in the Co region observed in the present study further supports this concept. Among individuals with facial asymmetry, the frontal occlusal plane often tilts in the same direction as the mandibular deviation [27]. According to Teng et al. [28], there is a statistically significant difference in the angle of the occlusal plane between individuals with mandibular deviation and those with symmetry. Their findings indicate that the angle of the occlusal plane is positively correlated with the degree of mandibular deviation; greater mandibular deviation is linked to greater inclination of the occlusal plane. Chen et al. [26] found that the distance from the proximal cusp of the maxillary first molar to the horizontal plane was longer on the nondeviated side among individuals with a deviated chin. This finding suggests that the midface area is longer on the unaffected side than on the deviated side.

Although maxillary sinus height cannot be directly equated with midface height or maxillary sinus height diameter, both of which depend on the height of the supporting maxillary bone, this trend may help explain the correlation between asymmetry in the Go area and maxillary sinus height observed in our study. Our results showed no correlation between left-right differences in maxillary sinus volume across all six asymmetry index items, suggesting that maxillary sinus volumes are not affected by jaw deviation. Ahn et al. [18], who studied skeletal class III individuals with facial asymmetry, also reported no differences in maxillary sinus volume between the sides, regardless of facial asymmetry status. According to the functional matrix theory of Moss and Salentijn [29], the development of the nasomandibular complex is driven by secondary and compensatory mechanical stimuli, such as nasal airflow, which promote vertical and lateral growth. It is possible that even when asymmetry occurs in the external craniofacial morphology, the internal structure (i.e., maxillary sinus) exhibits a compensatory mechanism to maintain balance.

Our results showed no significant differences in maxillary sinus volume among groups classified according to anteroposterior skeletal morphology (skeletal classes I, II, and III). However, in the study by Shrestha et al. [15], the skeletal class II group had a larger maxillary sinus volume compared with the skeletal class III group. When comparing the findings by Shrestha et al. [15] with our results, we noted differences in both anteroposterior and vertical maxillofacial skeletal morphology classifications. Whereas Shrestha et al. [15] observed no significant differences between groups based on vertical maxillofacial skeletal morphology, we found that the vertical group had significantly greater maxillary sinus height and volume than the average group, based on classification according to vertical skeletal relationships. This discrepancy may be due to differences in participant selection criteria or evaluation methods. Specifically, our study classified vertical maxillofacial skeletal morphology using the FMA angle, whereas Shrestha et al. [15] used the angle between the S-N plane and the Go-gnathion line. The study by Shrestha et al. [15] showed that there were no significant differences among the groups, but there was a trend toward larger maxillary sinus volume in the high-angle group.

In this regard, the trend aligns with our finding that the vertical group in our classification has a larger maxillary sinus volume than the average group. Vertical growth patterns may influence maxillary sinus development. For instance, vertical facial growth is characteristic of the vertical group and may promote vertical expansion of the maxillary sinus. The observation that the vertical group, as classified by vertical maxillofacial skeletal morphology, has significantly greater maxillary sinus height and volume than the average group suggests that vertical growth plays a role in maxillary sinus development. Specifically, vertical facial growth may drive the vertical expansion of the maxillary sinus. Although the mechanism underlying maxillary sinus growth is not fully understood, it has been suggested that the volume and morphology of the maxillary sinus serve as a compensatory mechanism to help maintain facial symmetry. We observed a correlation between the Co area and maxillary sinus length, as well as between the Go area and maxillary sinus height; no correlation was present regarding volume. Atul Kumar et al. [11] measured maxillary sinus height at three sites: anterior, central, and posterior. They found that the smallest maxillary sinus height values were located in the anterior part of the sinus (between the first and second molars) in the vertical group. In contrast, the largest values were evident in the posterior part of the maxillary sinus (also between the first and second molars) in the vertical group.

The maxillary sinus is considered to develop from birth to 18 years of age; however, Ariji et al. [30] reported that maxillary sinus volume continues to increase until the age of 20 years, after which it begins to decrease.

Similarly, Jun et al. [3] stated that maxillary sinus volume peaks between the ages of 21 and 30 years in men and between 11 and 20 years in women, then gradually declines. These studies indicate that the maxillary sinus size increases during growth, reaching a maximum size and then shrinking with age. In this study, we used CBCT data from 154 patients aged 18 to 38 years to investigate the correlation between maxillofacial asymmetry and maxillary sinus morphology; we sought to exclude the effects of growth and aging. However, the long-term morphological changes of the maxillary sinus remain unclear and warrant further investigation.

An understanding of the detailed characteristics of the 3D size of the maxillary sinus is beneficial for orthodontic treatment, particularly in cases involving orthognathic surgery, because it is closely related to craniofacial morphology. Although the lack of clear evidence persists regarding the correlation between facial asymmetry and maxillary sinus size, the present results may provide some insights. Importantly, because of the retrospective design, the statistical significance of our findings may have been influenced by several confounding factors related to sample selection and pooling. Future studies with larger sample sizes and a focus on sex differences are needed to provide more statistically robust results. Such studies could provide deeper insights into facial morphology and treatment planning, as well as a clearer understanding of the relationship between facial asymmetry and the maxillary sinus; this information is important for both research and clinical practice.

## Conclusions

This study found no left-right differences in maxillary sinus height, width, length, or volume based on linear measurements. However, positive correlations were observed between the facial asymmetry index at Go and the difference in maxillary sinus height and between the facial asymmetry index at Co and the difference in maxillary sinus length between the left and right sides. No correlation was present between the facial asymmetry index and the difference in maxillary sinus volume between the left and right sides.

Nevertheless, statistically significant differences were noted among the three groups based on their vertical maxillofacial skeletal morphology, with the vertical group showing greater maxillary sinus height and volume than the average group on both the right and left sides. Asymmetry in specific facial regions may relate to regional variations in maxillary sinus morphology, offering insights into the link between facial asymmetry and maxillary sinus structure.
